# Longitudinal profiling of acute phase and cytokine responses in sheep supplemented with lemon essential oil during summer season

**DOI:** 10.3389/fvets.2026.1837990

**Published:** 2026-06-12

**Authors:** Maria Giovanna Ciliberti, Mariangela Caroprese, Marialetizia Ponte, Agostino Sevi, Marzia Albenzio

**Affiliations:** 1 Department of Agriculture, Food, Natural Resources, and Engineering (DAFNE), University of Foggia, Foggia, Italy; 2 Department of Agricultural, Food and Forestry Sciences, University of Palermo, Palermo, Italy

**Keywords:** acute phase response, chronic stress, feed additives, inflammation, lemon essential oil, terpenes

## Abstract

Heat stress (HS) represents a significant environmental stressor for small ruminant production occurring during summer season, which affects feed intake, physiological homeostasis, immune function, and overall performance. Nutritional strategies based on functional feed additives, including essential oils (EO), have been proposed to enhance thermotolerance and mitigate the negative effects of HS. This study investigated whether dietary integration of lemon EO, supplied either as free oil or in microencapsulated form at two dosages, can modulate the acute phase response in sheep exposed to HS during summer season. Acute phase proteins (C-reactive protein, haptoglobin, ceruloplasmin, and albumin) and pro-inflammatory cytokines (TNF-α, IL-1β, IL-6) was monitored for 42 days to characterize temporal dynamics of systemic inflammatory and stress responses connected with physiological responses during HS. Forty balanced dairy sheep were allocated to five dietary treatments: a control diet (CON), high-dose lemon EO (HEO, 800 mg/day), low-dose lemon EO (LEO, 400 mg/day), high-dose microencapsulated lemon EO (HEOM, 800 mg/day), and low-dose microencapsulated lemon EO (LEOM, 400 mg/day) and individually fed with a concentrate diet based on corn (50%), soybean meal (20%), barley (15%), beet pulp (12.5%), and a fat supplement (2.5 %). Blood samples were collected on days 0, 14, 28, and 42, and on plasma the concentrations of acute phase proteins (C-reactive protein-Crp, Haptoglobin-Hp, Ceruloplasmin-Cp, and Albumin-Alb) and cytokines (TNF-α, Il-6, and IL-1β) were determined by ELISA. Results showed that LEO and HEOM groups showed higher Hp concentrations than the CON group after 28 days of supplementation. Moreover, the LEOM group exhibited higher Cp levels than the HEOM group after 14 days. Albumin concentration was the highest in the CON group at day 14, whereas at day 28 it exhibited the opposite trend. Cytokine profile displayed a reduced level of TNF-α in lemon EO supplemented experimental groups, while the IL-1β level increased overall from the beginning to the end of the experiment with lower level registered in HEOM group than the CON and LEOM groups. Present data demonstrated that lemon EO influenced the acute phase protein and cytokine responses, indicating a modulatory effect on systemic adaptation to prolonged heat load during summer season.

## 1 Introduction

Heat stress (HS) is one of the major challenges affecting for small ruminant production worldwide. Prolonged exposure to high ambient temperatures often results in decreased feed intake, impaired growth performance, reduced reproductive success, and overall compromised welfare and productivity (1). Small ruminants respond to thermal stress through behavioral, physiological, and metabolic adaptations to maintain homeothermy. However, when heat load is prolonged, especially under conditions of high humidity, solar radiation, and poor ventilation, these adaptive mechanisms become insufficient, leading to negative effects on health and productivity (2).

In addition to thermoregulatory responses, HS is increasingly recognized as a condition that induces systemic inflammation and oxidative stress. These processes are associated with alterations in circulating metabolites and proteins, indicating that the animals' response to thermal challenge involves not only thermoregulation but also immune and inflammatory pathways as extensively reviewed by Most and Yates (3). Despite this evidence, most studies in small ruminants have primarily focused on productive traits, physiological parameters (e.g., respiration rate, body temperature) (4) or molecular markers such as heat shock proteins (5), with less attention given to integrated inflammatory responses (6, 7).

Body temperature (BT) is widely used as an indicator of thermal stress, reflecting the activation of neuroendocrine and thermoregulatory mechanisms (8). Under HS conditions, animals may develop stress-induced hyperthermia (9). However, chronic exposure to heat not only affects thermoregulation but also disrupts immune and biochemical homeostasis, promoting systemic inflammatory responses (10).

Nutritional strategies have been proposed as potential tools to mitigate the adverse effects of HS and enhance animal resilience (15). Feed additives, including essential oils (EOs), vitamins, and other functional compounds, may support metabolic balance and reduce oxidative and inflammatory stress (11, 12). Essential oils, in particular, have been shown to improve rumen fermentation, feed efficiency, and product quality (13). Such an example, a mixtures based on anise, clove, and thyme EO supplementation have been found to enhance nutrient digestibility and increase milk fat concentration and unsaturated fatty acids without affecting intake or milk yield in goats (16). Additionally, sheep supplemented with microencapsulated mixture of cinnamaldehyde, eugenol, carvacrol, and capsicum oleoresin have reduced methane production, indicating potential benefits for rumen fermentation and environmental impact (17). However, their effects are inconsistent, likely due to variability in chemical composition and formulation (14). Moreover, limited information is available on their role in modulating systemic inflammation mediated by cytokines, and downstream systemic responses activated by acute phase protein-APP secretion, especially under HS conditions.

We hypothesized that lemon EO supplementation, particularly in microencapsulated form, would mitigate HS-induced inflammation by modulating cytokine production and APP profiles, thereby improving thermotolerance. Therefore, the aim of this study was to evaluate the effect of dietary supplementation with lemon essential oil, including a microencapsulated formulation, on the temporal dynamics of selected APPs (C-reactive protein, haptoglobin, ceruloplasmin, and albumin) and pro-inflammatory cytokines (TNF-α, IL-1β, IL-6) in sheep exposed to heat stress during the summer season.

## 2 Material and methods

### 2.1 Animals and experimental design

Forty Valle del Belice, a Sicilian dairy sheep breed known for its adaptability to Mediterranean environments and good milk production used for producing raw-milk traditional cheeses (18), were enrolled in the study. Primiparous dairy sheep were balanced for body condition score (BCS; 3.01 ± 0.05), milk production (1,110 ± 10.5 g/day per head), stage of lactation (DIM; 81 ± 0.26 days), and body weight (BW; 40.2 ± 0.07 kg), and then randomly assigned to five experimental groups receiving dietary treatments based on lemon EO as feed additives. The control (CON) group received a conventional diet (*n* = 8); the HEO and LEO groups received high (800 mg/day, *n* = 8) or low (400 mg/day, *n* = 8) doses of EO, respectively; the HEOM and LEOM groups received high or low doses of microencapsulated EO for rumen protection (800 and 400 mg/day, respectively *n* = 8 for each; 1 g microencapsulated product contained 0.172 g EO). The chemical composition of lemon EO was characterized by a high content of d-limonene (91.20%). Microencapsulation of lemon EO was performed as previously reported in Maggiolino et al. (19), by using a lemon EO-in-water emulsion and alginate, with a 30% core–shell ratio in Endraguard^®^. Microencapsulated lemon EO supplements were mixed with 50 g of concentrate and top-dressed individually onto the TMR once daily at 07:00 h. The concentrate consisted of corn (50%), soybean meal (20%), barley (15%), beet pulp (12.5%), and ZOOFAT (2.5%, ZOOTEAM SRL, Palermo, Italy), containing 17.8% crude protein, 4.1% ether extract, 13.8% NDF, 6.2% ADF, 0.6% ADL, and 3.4% ash on a DM basis. Polyphite hay was offered *ad libitum* and contained 7.3% crude protein, 1.7% ether extract, 59.9% NDF, 43.4% ADF, 5.6% ADL, and 9.2% ash on a DM basis. Water was provided *ad libitum*. The animals consumed the entire amount of feed offered (800 g/d of concentrate). The experimental trial lasted 42 days (June–August 2024), including 1 week of diet adaptation. All sheep were housed in the same pen. Average milk yield during the experimental trial was 1,068.8 ± 19.22 g/day and did not differ among experimental groups. The study protocol was approved by the Animal Welfare Committee of the University of Palermo (protocol number 201948-12-12-2023).

### 2.2 Air temperature and relative humidity data collection

Two thermo-hygrometers (PCE-WBGT 10, PCE Instruments, Meschede, Germany) were placed inside and outside the shed placed at height of 90 cm to record environmental temperature and relative humidity throughout the experimental trial. Data from thermo-hygrometers were collected every 15 min. Measures of air temperature (°C) and relative humidity (%) were combined into temperature humidity index (THI) by using the following Kelly and Bond (20) formula *THI* = (1.8 × *Tdb*+32)−(0.55 − 0.005 × *RH*) × (1.8 × *Tdb*−26), where Tdb represents the dry-bulb temperature expressed in degrees Celsius (°C), and RH represents the relative humidity expressed as a percentage (%). Additionally, skin temperature (ST) was continuously monitored using a Digitanimal GPS collars (DIGITANIMAL, S.L.^®^, https://digitanimal.com) which integrates IoT sensors and a surface temperature sensor. ST from each animal were recorded every 30 min.

### 2.3 Blood sample collection

Blood samples (7 mL) were obtained via jugular venipuncture on days 0, 14, 28, and 42 using lithium-heparinized Vacutainer tubes, as lithium heparin is considered suitable for cytokine and soluble protein determination (59). Within 1–2 h after collection, blood samples were centrifuged at 1,500 *g* for 15 min at room temperature, and the plasma obtained was collected and stored at −20 °C until analysis by ELISA.

### 2.4 Acute phase proteins determination

Plasma levels of acute phase proteins were assessed using commercial ELISA kits specific for sheep, including those for C-reactive protein (A303869, Antibodies, Stockholm, Sweden), haptoglobin (Hp; MBS8820035, Mybiosources, San Diego, USA), ceruloplasmin (Cp; MBS8820034, Mybiosources, San Diego, USA), and albumin (Alb; MBS1602124, Mybiosources, San Diego, USA). All assays were performed in duplicate for each animal following the instructions provided by the respective manufacturers.

### 2.5 Cytokines determination by ELISA

All the assays were optimized in our laboratory for concentrations of mouse monoclonal antibodies (mAb), supernatants, polyclonal detecting antibody (Ab) and secondary conjugate Ab. For IL-6 and IL-1β quantification was followed the procedure described by Ciliberti et al. (21). Briefly, specific mouse monoclonal antibodies against ovine IL-6 and IL-1β (Biorad, Hercules, CA, USA) were used as capture antibodies, and rabbit polyclonal anti-sheep IL-6 and IL-1β antibodies (Biorad, Hercules, CA, USA) were used as detection reagents. All incubations were performed at 37 °C which increase the rate of reaction between antibody and antigen as previously reported for cytokine ELISA optimization (22). Cytokine concentrations were expressed in nanograms per milliliter (ng/mL) by comparing the samples to a standard curve prepared using serial dilutions of recombinant ovine IL-6 (Cusabio Biotech Co., Wuhan, P.R. China) and recombinant ovine IL-1β (Kingfisher Biotech Inc., St. Paul, MN, USA).

TNF-α levels were determined using an ovine TNF-α polyclonal antibody (Kingfisher Biotech Inc., St. Paul, MN, USA; final concentration 2 μg/mL) as the capture antibody and a biotinylated ovine TNF-α polyclonal antibody (Kingfisher Biotech Inc., St. Paul, MN, USA; final concentration 1 μg/mL) as the detection antibody. Streptavidin–HRP conjugate (Biorad, Hercules, CA, USA; diluted 1:500 in PBS) was used as the secondary antibody. All incubations were performed at room temperature. A recombinant ovine TNF-α protein (Kingfisher Biotech Inc., St. Paul, MN, USA) was used to build the standard curve, and concentrations were expressed in ng/mL. All measurements were performed in duplicate for each animal. The intra-assay coefficients of variation (CV) were 9.4 % for IL-1β, 10% for IL-6, and 9.7% for TNF-α, respectively. Limit of detection for the was 109.38 ng/mL IL-6, 15.63 ng/mL for IL-1β, and 156.25 ng/mL for TNF-α, respectively.

### 2.6 Statistical analysis

The G^*^Power package was used to calculate sample size achieving a power >0.80 with an α = 0.05 (23). All variables were checked for normality prior to analysis. When this assumption was not satisfied, a log10 transformation was performed. All data were analyzed using the MIXED procedure of SAS (58). The statistical model included the fixed effects of dietary strategy (CON, HEO, LEO, HEOM, LEOM), sampling time (0, 14, 28, and 42 days), and their interaction. Individual animals were included as a random effect. For skin temperature daily mean values registered among the experimental 6 weeks were applied as sampling time fixed effect. When significant effects were detected, Tukey's *post hoc* test was applied to adjust for multiple comparisons among dietary treatments, sampling time, and their interaction. Meteorological data were explored in terms of mean, maximum and minimum, standard error values by SAS software. Person correlation analysis was performed to assess the correlation between daily mean values skin temperature and meteorological data (THI inside and outside, and air temperature inside and outside). A simple linear regression was then performed in order to study the relationship between a single dependent variable (skin temperature, ST) and the independent variables, denoted by *X* (indoor air temperature, AT). All data are expressed as least square mean ± SEM. Statistical significance was declared at *P* < 0.05. Figures and linear regression were made with GraphPad Prism Software version 10.0 (San Diego, CA, USA).

## 3 Results

### 3.1 Meteorological data and skin temperature

**Figure 1:**
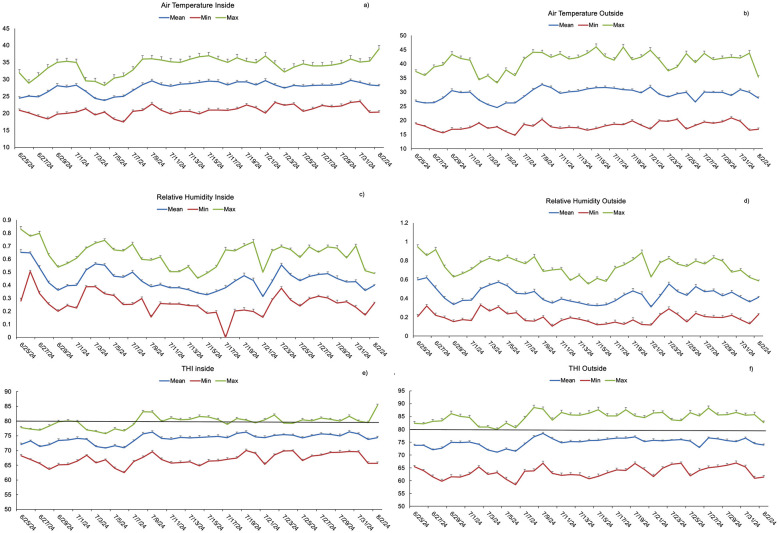
Meteorological data measured throughout the experimental trial in terms of air temperature recorded **(a)** inside and **(b)** outside the shed, relative humidity recorded **(c)** inside and **(d)** outside, and of temperature humidity index (THI) **(e)** inside and **(f)** outside.

**Figure 2:**
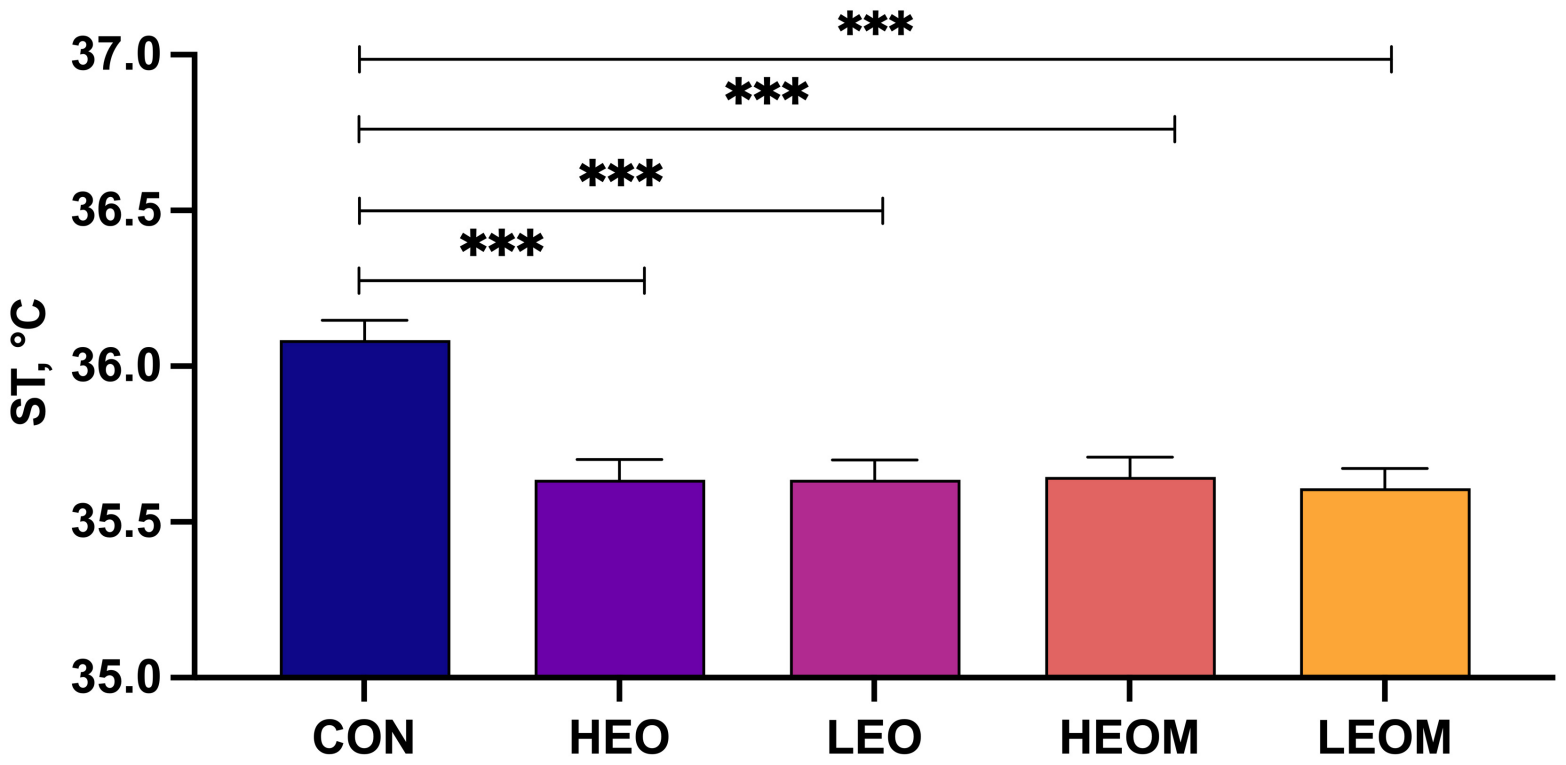
Least square means ± SEM of skin temperatures temperature measured with GPS collar equipped temperature biosensor in sheep receiving control (CON) diet, a feed additive based on: lemon essential oil at high dose (HEO), lemon essential oil at low dose (LEO), microencapsulated lemon essential oil at high dose (HEOM), and microencapsulated lemon essential oil at low dose (LEOM), respectively under summer season. Bar with asterisks indicated significant differences among experimental groups (***P < 0.0.01).

**Figure 3:**
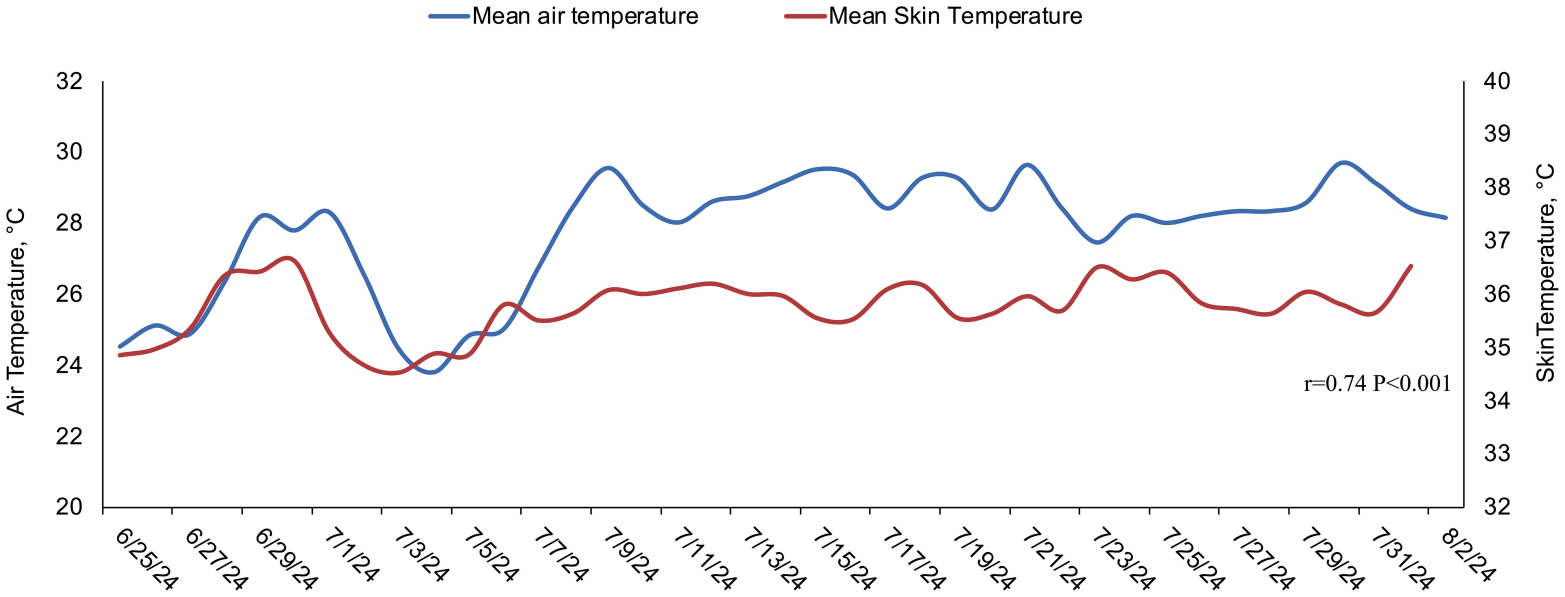
Time series plot of average temperatures collected all day during on same dates for the environmental temperature and the skin temperature measured with GPS collar equipped temperature biosensor.

**Figure 4:**
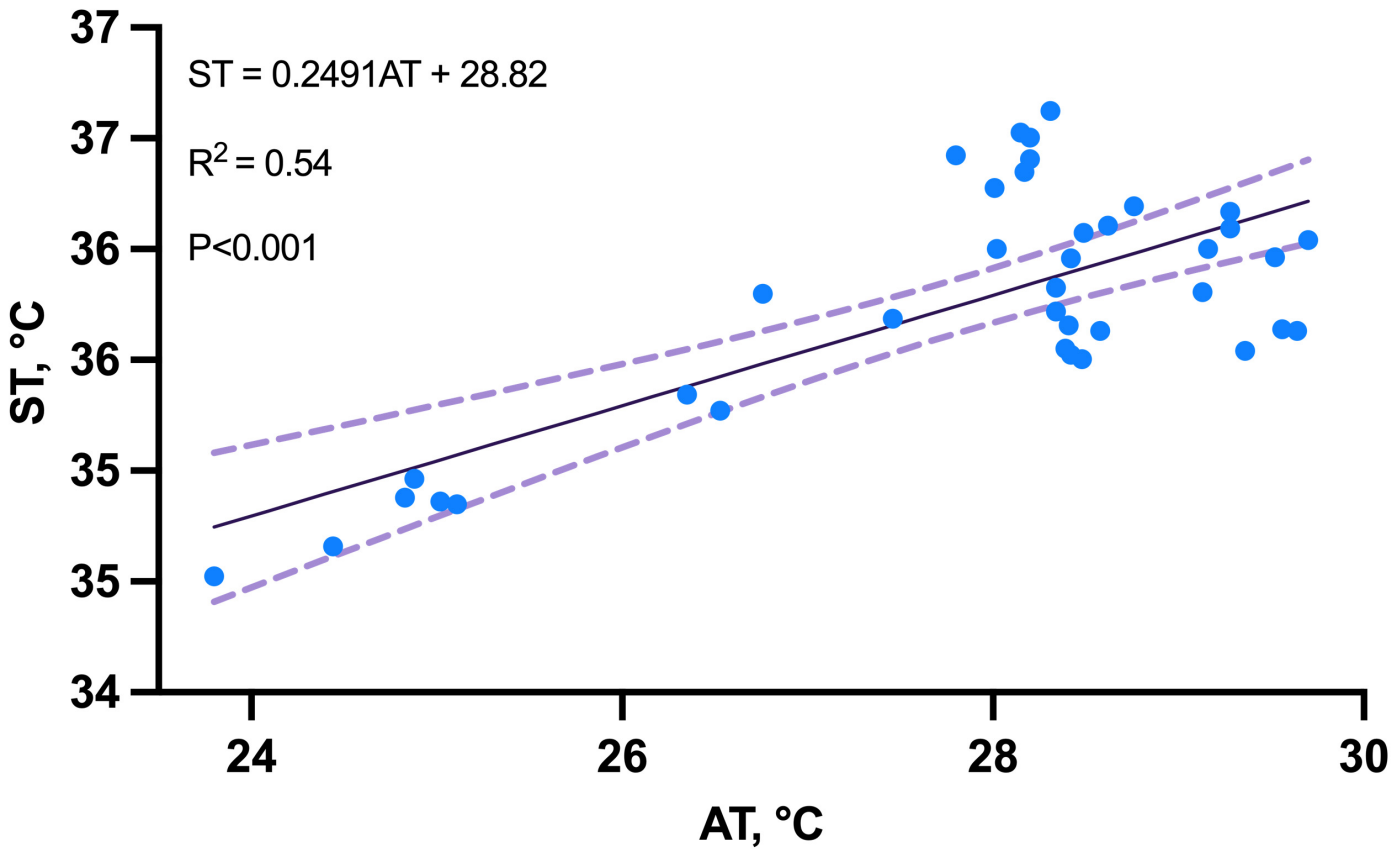
Simply linear regression between skin temperature (ST) and air temperature (AT).

Figure 1 reports air temperature (AT) recorded inside and outside (Figure 1a), relative humidity (Figure 1b) and THI (Figure 1c). The skin temperature (ST) measured using GPS collars equipped with temperature biosensors, was significantly affected by dietary strategy (P < 0.001, Figure 2), and sampling time (*P* < 0.001). A significant decrease in all EO supplemented groups, regardless of dosage or microencapsulation procedure, compared to the CON group was registered. Moreover, the second experimental week showed, on average, the lowest skin temperature (35.03 °C), whereas the highest values were recorded during the third, fifth, and sixth weeks (35.98, 35.75, and 36.03 °C, respectively). Pearson correlation analysis among meteorological data and ST showed a strong correlation between ST and indoor AT (*r* = 0.74, *P* < 0.001; Figure 3) and a moderate correlation with outdoor AT (*r* = 0.57, *P* < 0.001). Similarly, mean ST was moderately associated with THI values, with a higher correlation observed for indoor THI (*r* = 0.63, *P* < 0.001) compared to outdoor THI (r = 0.53, *P* < 0.001). Given the stronger correlation between ST and indoor AT, linear regression was performed using indoor AT as the predictor. The analysis revealed a significant positive association, described by the equation *Y* = 0.2491*X* + 28.82. The slope was 0.2491 ± 0.0383 (95% CI: 0.1715–0.3268), indicating that for every 1 °C increase in air temperature, ST increased by approximately 0.25 °C. The slope was significantly different from zero (*F* = 42.32; DF = 1, 36; *P* < 0.0001). The intercept was 28.82 ± 1.07 (95% CI: 26.65–30.98). The model explained 54% of the variation in ST (*R*^2^ = 0.54, Figure 4), with a standard error of the estimate of 0.37. The reciprocal of the slope (1/slope = 4.014) indicates that approximately 4 °C of change in air temperature is required to induce a 1 °C change in ST.

### 3.2 Acute phase proteins

**Figure 5:**
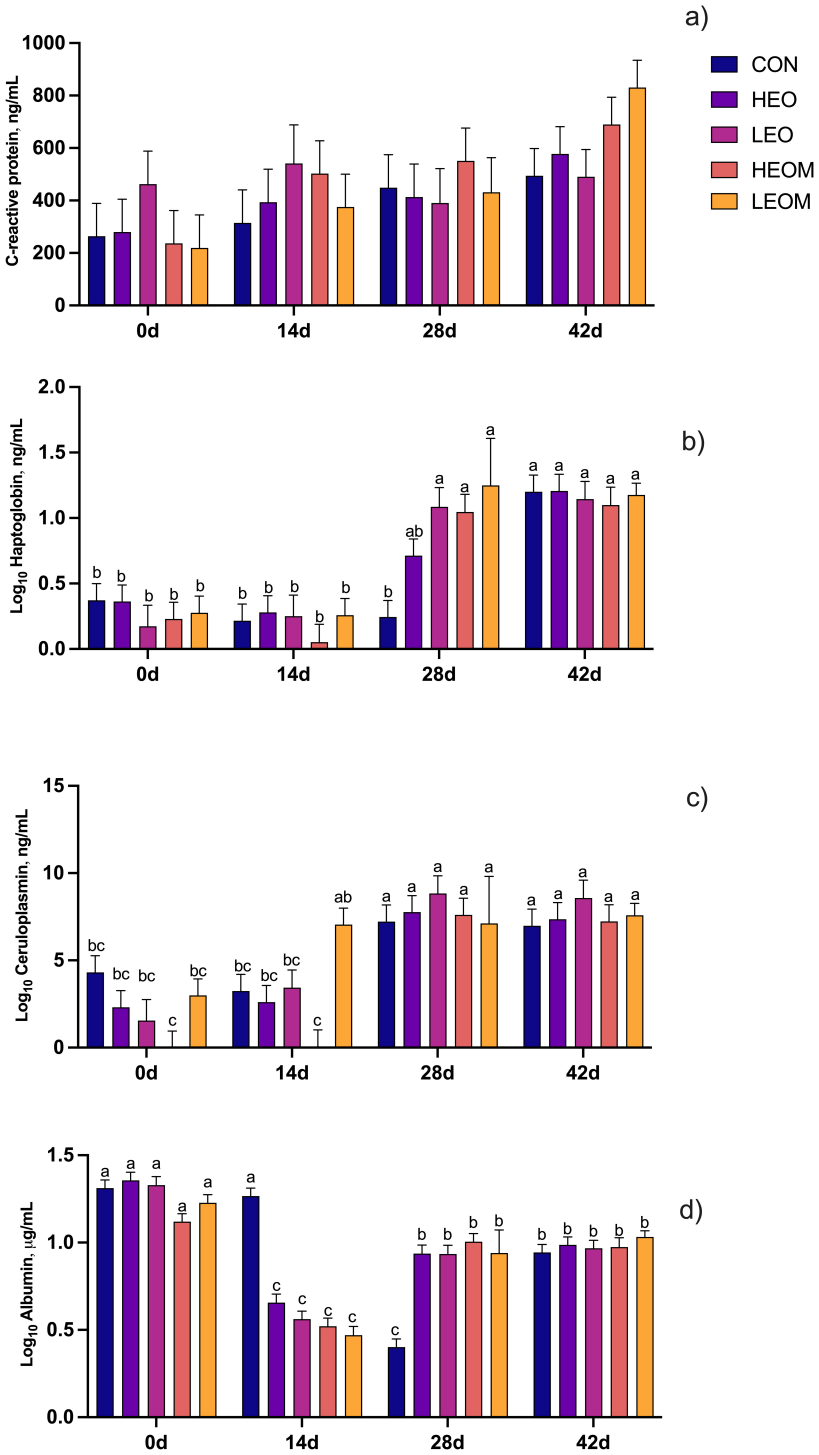
Least square means ± SEM of acute phase proteins measured in terms of **(a)** C-reactive protein **(b)** haptoglobin, **(c)** ceruloplasmin, and **(d)** albumin at 0, 14, 28 and 42 days of the trial in plasma of sheep receiving control (CON) diet, a feed additive based on: lemon essential oil at high dose (HEO), lemon essential oil at low dose (LEO), microencapsulated lemon essential oil at high dose (HEOM), and microencapsulated lemon essential oil at low dose (LEOM), respectively under summer season. ^a, b, c^Values with different letters differ between feeding treatments within a sampling day (*P* < 0.05).

Temporal changes in acute phase protein levels for the different experimental groups are presented in Table 1. C-reactive protein levels were significantly affected by sampling time (*P* = 0.0002), showing an increase at the end of the experiment compared with both the beginning and day 14 (Table 1). No significant effects were observed for dietary strategy or for its interaction with sampling time (Figure 5a). The value of Hp was significantly influenced by time of sampling (*P* < 0.001), and the interaction between dietary strategy and time of sampling (*P* = 0.009), increasing on average after 28 days of the experiment and remaining stable until the end of the experiment (Table 1). In particular, the LEO and HEOM groups showed higher Hp levels than the CON group after 28 days of supplementation, while the CON group reached comparable levels only after 42 days (Figure 5b)

**Table 1:** Effect of time of sampling and dietary strategy on acute phase proteins measured in terms of C-reactive protein, haptoglobin, ceruloplasmin, and albumin in plasma of sheep under summer season.

Acute phase proteins	Time of sampling				SEM	Dietary strategy					SEM	*P-*value		
	0d	14d	28d	42d		CON	HEO	LEO	HEOM	LEOM		*T*	*D*	*T* ^*^ *D*
CRP, ng/mL	292.430^b^	425.440^b^	447.100^a^	616.370^a^	54.488	171.320	138.620	163.370	214.970	171.000	18.657	0.0002	0.677	0.597
Hp, Log ng/mL	0.2829^c^	0.2117^c^	0.8675^b^	1.1656^a^	0.067	0.508	0.641	0.664	0.607	0.740	0.074	< 0.001	0.305	0.009
Cp, Log ng/mL	2.235^c^	3.266^c^	7.709^b^	7.547^a^	0.491	5.439^ab^	5.013^ab^	5.599^ab^	3.711^b^	6.185^a^	0.549	< 0.001	0.025	0.025
Alb, Log 6g/mL	1.269^a^	0.695^d^	0.844^c^	0.981^b^	0.024	0.981	0.984	0.948	0.905	0.918	0.027	< 0.001	0.101	< 0.001

CRP, C-reactive protein; HP, haptoglobin; Cp, ceruloplasmin; Alb, albumin; *T*, time of sampling; *D*, dietary strategy; CON, control group; HEO, lemon essential oil at high dose supplemented group; LEO, lemon essential oil at low dose supplemented group; HEOM, microencapsulated lemon essential oil at high dose supplemented group; LEOM, microencapsulated lemon essential oil at low dose supplemented group; Cp, ceruloplasmin; Hp, haptoglobin; Alb, albumin.

^a, b, c, d^Values within a row with different superscripts differ significantly at *P* < 0.05.

Ceruloplasmin (Cp) levels were significantly affected by dietary strategy (*P* = 0.025), sampling time (*P* < 0.001, Table 1), and the interaction between dietary strategy and sampling time (*P* = 0.025). On average, the HEOM group had lower Cp levels than the LEOM group (Table 1). Additionally, Cp levels increased at 28 days and remained high until the end of the experiment (Table 1). Moreover, after 14 days, the LEOM group exhibited higher Cp levels than the HEOM group, as well as compared to the values recorded at the beginning of the trial (Figure 5c).

Time of sampling (*P* < 0.001) and the interaction between dietary strategy and time of sampling (*P* < 0.001), significantly influenced the levels of Alb. Over the course of the experiment, Alb levels were lower at day 14 than at the beginning, then gradually decreased remaining below the initial values (Table 1). In particular, the CON group showed the highest Alb concentration at day 14, whereas at day 28 it exhibited the opposite trend, showing lower levels than the supplemented lemon EO groups (Figure 5d).

### 3.3 Cytokine profile

**Figure 6:**
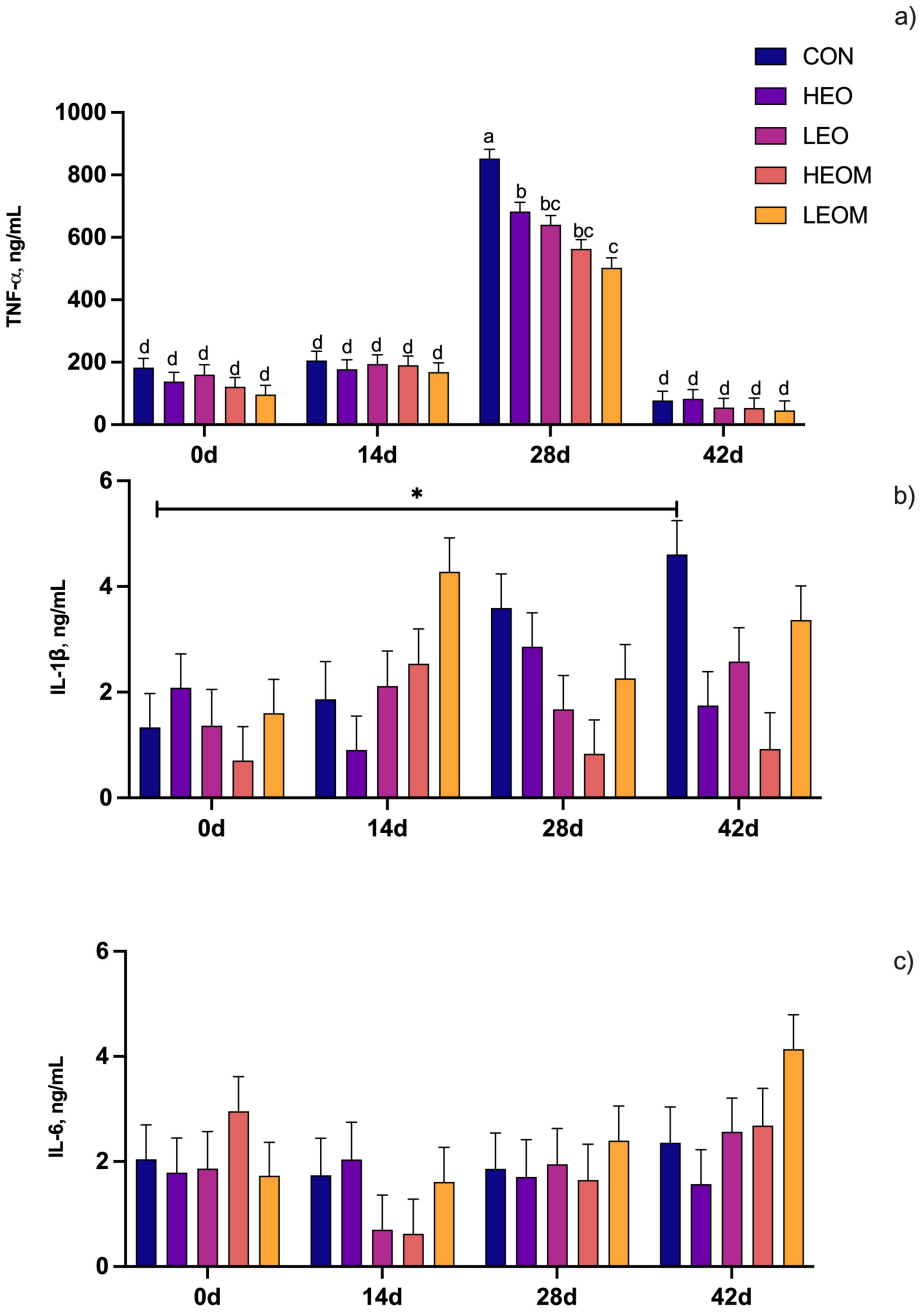
Least square means ± SEM of cytokine profile measured in terms of **(a)** TNF-α, **(b)** IL-1β, and **(c)** IL-6 measured in plasma at day 0, 14, 28 and 42 of the trial in sheep receiving control (CON) diet, a feed additive based on: lemon essential oil at high dose (HEO), lemon essential oil at low dose (LEO), microencapsulated lemon essential oil at high dose (HEOM), and microencapsulated lemon essential oil at low dose (LEOM), respectively under summer season. Bar with asterisks indicated significant differences among experimental groups (*P < 0.05). ^a, b, c, d^Values with different letters differ between feeding treatments within a sampling day (*P* < 0.05).

Cytokine concentrations measured at each sampling time in the experimental groups are summarized in Table 2. TNF-α concentrations were significantly influenced by sampling time (*P* < 0.001), dietary strategy (*P* < 0.001), and their interaction (*P* < 0.001). Overall, the CON group exhibited the highest TNF-α levels compared with all other dietary treatments, whereas the LEOM group showed lower concentrations than both the LEO and HEO groups (Table 2). Across sampling times, TNF-α levels increased after day 28 and subsequently declined to values lower than those recorded at the beginning of the experiment (Table 2). The interaction effect indicated that lemon EO supplementation lowered TNF-α concentrations after day 28 compared with the CON group (Figure 6a). The IL-1β concentrations increased overall from the beginning to the end of the experiment (*P* = 0.022, Table 2). The HEOM group had significantly lower IL-1β levels than the CON and LEOM groups (*P* = 0.001, Table 2). Furthermore, a significant interaction effect (*P* = 0.0067) revealed that IL-1β concentrations in the CON group were higher at day 42 than the beginning of the experiment (Figure 6b). IL-6 concentrations were significantly influenced by sampling time (*P* = 0.02, Table 2), increasing from day 14 to day 42, while neither dietary strategy nor the interaction between diet and time affected IL-6 levels (Figure 6c).

**Table 2:** Effect of time of sampling and dietary strategy on cytokine profile measured in terms of IL-6, IL-1β, and TNF-α secreted in plasma of sheep under summer season.

Cytokines level	Time of sampling				SEM	Dietary strategy					SEM	*P-*value		
	0d	14d	28d	42d		CON	HEO	LEO	HEOM	LEOM		*T*	*D*	*T* ^*^ *D*
TNF-α, ng/mL	139.620^c^	187.210^b^	648.280^a^	62.852^d^	13.391	329.640^a^	270.580^b^	262.390^b^	232.060^bc^	202.780^c^	15.052	< 0.001	< 0.001	< 0.001
IL-1β, ng/mL	1.419^b^	2.341^ab^	2.246^ab^	2.6454^a^	0.291	2.851^a^	1.899^ab^	1.934^ab^	1.252^b^	2.878^a^	0.329	0.022	0.001	0.007
IL-6, ng/mL	2.075^ab^	1.3429^b^	1.912^ab^	2.6621^a^	0.301	1.999	1.776	1.766	1.979	2.469	0.341	0.023	0.563	0.471

*T*, time of sampling; *D*, dietary strategy; CON, control group; HEO, lemon essential oil at high dose supplemented group; LEO, lemon essential oil at low dose supplemented group; HEOM, microencapsulated lemon essential oil at high dose supplemented group; LEOM, microencapsulated lemon essential oil at low dose supplemented group.

^a, b, c, d^Values within a row with different superscripts differ significantly at *P* < 0.05.

## 4 Discussion

### 4.1 Experimental environmental condition

Recently, aromatic plants and their EOs have been widely applied as feed additives in animal nutrition due to current restrictions in the use of antibiotics (24, 25). Additionally, EOs may contribute to improving animal health and production performance (24, 26). The biological activity of EOs depends on their chemical composition, plant origin, inclusion level, and pharmacokinetics (24, 27). Several EOs have been found to promote multiple immunomodulatory actions, especially when animals are in immune-suppressed conditions. Notably, no studies have evaluated dietary lemon EO effects on the acute phase response in sheep during the summer season. In the present experiment, THI measured inside the shed, which was highly correlated with ST, ranged between 67 and 80. Moreover, the ST measured by GPS collars ranged between 35.75 and 41.62 °C. Those values are in line with rectal temperatures found by Sevi et al. (28), who suggested that prolonged exposure to maximum air temperatures over 30 °C and a THI of 80 impair the thermal balance of dairy sheep and induce HS. Under high ambient temperatures, changes in body temperature, respiration rate, and heart rate can be used to measure an animal's reaction to HS (29). A relevant aspect of this study is that skin temperature was monitored through GPS collars rather than through intermittent rectal measurements, which is considered invasive and time-consuming. Moreover, to measure RT induce severe stress to animal, increasing metabolic heat production associated with the flight response (9). The lemon EO supplemented groups showed an approximately 1% reduction in ST compared to the control. These skin temperature trends suggest improved thermoregulatory efficiency in lemon EO supplemented groups. Moreover, Pearson correlation analysis indicated that environmental THI was strongly associated with skin temperature.

### 4.2 Acute phase response: acute phase protein secretion

Heat stress is considered a non-infectious stressor (30) that can trigger a systemic acute phase response in ruminant (31), characterized by the hepatic synthesis and release of APPs, such as CRP, serum amyloid A (SAA), and Hp (32). These proteins are recognized as useful stress biomarkers in cattle and several other species (33–37). The magnitude of APP response varies widely depending on the protein and the stressor (32). CRP is a major APP belonging to the pentraxin family, involved in activation of the complement system and immune modulation (38). The physiological behavior of CPR during infectious or non-infectious diseases in sheep and goats are not fully elucidated; however, its levels were found to increase during pneumonia infection (39). Despite lemon EO supplementation, CRP levels increased, this is in agreement with findings in goats, where CRP did not differ between animals exposed to direct solar radiation or shading (1), and in cows, which showed similar CRP concentrations across cold and hot seasons (40). Overall, CRP increased at day 28, confirming activation of the acute phase response under prolonged heat stress.

Among the APPs, Hp is considered a major acute-phase protein in livestock (41), which increases in response to inflammation and stress (42), as reported in goats during summer (1). Hp concentration increased also in the present experiment with kinetics similar to those of CRP. However, LEO and HEOM supplementation resulted in a more rapid rise compared with the other experimental groups, suggesting an effect of supplementation on the kinetics of the acute phase response. Thus, promoting timely APP secretion in a way consistent with an appropriate physiological activation of the acute phase response rather than a pathological inflammatory process. Moreover, individual variability in APP response has been reported and may contribute to differences in stress susceptibility (43), partly due to differences in the response kinetics of specific APPs, with Hp reacting more slowly than SAA (43, 44).

Only a limited number of studies have documented the kinetics of Cp during HS exposure. Cp is a copper-containing plasma APP with antioxidant properties (45) classified as a minor APP in sheep and goats (46). In the present experiment, Cp levels showed a progressive increase during the trial. Moreover, the microencapsulated lemon EO groups showed a dose-dependent trend, with the high concentration leading to higher Cp levels than the low concentration; however, this trend did not result in any differences with the other experimental groups. Several studies have reported increases in Cp associated with elevated oxidative stress markers in human disorders (47, 48) and during hyperthermia in rats (49). Therefore, the increase in Cp concentration under high ambient temperature may represent an adaptive response to increased reactive oxygen species production associated with HS exposure (5).

Alb is considered a negative APP together with transferrin and transthyretin, and represents a major source of amino acids used for the synthesis of positive APPs during the acute phase response (50, 51). Furthermore, the initiation of the acute phase response triggers a downregulation of Alb production (32), this negative trend explains the reduction observed in all experimental groups at day 14 compared with the CON group. However, due to its relatively long half-life, Alb can also be used as an indicator of nutritional status (51). At day 28, all supplemented groups showed increased Alb levels, which may reflect changes in protein metabolism during the experimental period. Overall, the results indicate that heat stress induced a consistent acute phase response, while dietary supplementation modulated the kinetics of selected APPs without altering the overall magnitude of the response.

### 4.3 Acute phase response: acute phase cytokines

Cytokine cascades are activated in response to inflammation or metabolic stress, leading to regulation of innate immunity and APP synthesis (52). TNF-α, IL-1β, and IL-6 act in a coordinated sequence, with IL-6 serving as the main inducer of hepatic acute phase proteins (53). The cytokine profile observed in this study aligns with the coordinated sequence typically described during inflammatory and HS challenges. TNF-α, typically an early cytokine in the inflammatory cascade, showed a peak at day 28 followed by a decline. Accordingly, in heat-stressed sheep, skeletal muscle expression of TNF-α increased (5). Moreover, in sheep, the level of TNF-α in plasma increased 42 d post-partum, and a dietary treatment based on polyunsaturated fatty acids from flaxseed reduced its value (54). In the present experiment, EO supplementation, particularly in microencapsulated form, was associated with lower TNF-α levels, suggesting a dampening of the initial pro-inflammatory response.

IL-1β, acting downstream of TNF-α, contributes to amplification of the inflammatory response and induction of IL-6 synthesis which itself is the principal mediator and regulator of hepatic synthesis of APPs (55). The lower level of IL-1β found in the microencapsulated formulation at the highest concentration, supports a modulatory effect of the lemon EO on cytokine amplification during HS. Indeed, after long HS exposition (at day 42) only in the CON group the level of IL-1β increased. This modulatory effect is consistent with previous findings reporting reduced pro-inflammatory cytokines following essential oil supplementation in poultry (56). IL-6, the main systemic mediator of the acute phase response, increased progressively from day 14 to day 42 regardless of diet, indicating that heat stress was the primary driver of its activation. This was consistent with the observed increase in APPs, confirming IL-6 as the central regulator of the systemic acute phase response. In ruminants, chronic HS has been shown to induce detrimental pro-inflammatory responses that can compromise animal productivity (57); therefore, a modulation of proinflammatory responses by EO can maintain metabolic spare for animal productivity. Overall, cytokine pattern indicates that EO supplementation mainly modulated the early stages of the inflammatory cascade attenuating TNF-α and IL-1β responses, while the IL-6-mediated acute phase pathway remained largely unaffected under HS. However, some limitations should be acknowledged, including the relatively limited sample size, the focus on a single seasonal HS period, and the use of a specific breed (Valle del Belice sheep), which may limit the generalization of the findings.

## 5 Conclusions

To our knowledge, no previous studies have characterized the acute phase response in sheep exposed to HS during summer in terms of both acute phase proteins and pro-inflammatory cytokines. Results from the present study indicate that EO supplementation during HS can modulate the production of acute phase proteins and early cytokines of the TNF-α/IL-1β cascade by altering their temporal dynamics.

Future research should investigate the long-term effects of EO supplementation across different climatic conditions and breeds, as well as its impact on productive and reproductive performance, gut microbiota, and molecular pathways involved in stress adaptation. This may contribute to improved resilience and welfare in livestock systems exposed to increasing environmental temperatures due to climate change.

## Data Availability

The raw data supporting the conclusions of this article will be made available by the authors, without undue reservation.
